# The effect of prophylactic hemoclip placement and risk factors of delayed post-polypectomy bleeding in polyps sized 6 to 20 millimeters: a propensity score matching analysis

**DOI:** 10.1186/s12876-020-01454-1

**Published:** 2020-09-22

**Authors:** Chun-Wei Chen, Chia-Jung Kuo, Cheng-Tang Chiu, Ming-Yao Su, Chun-Jung Lin, Puo-Hsien Le, Siew-Na Lim, Chau-Ting Yeh, Malcolm R. Alison, Wey-Ran Lin

**Affiliations:** 1 Department of Gastroenterology and Hepatology, Linkou Chang Gung Memorial Hospital, No 5, Fu Hsing Street, Guishan Dist., Taoyuan, 333 Taiwan; 2 College of Medicine, Chang Gung University, Taoyuan, Taiwan; 3 Department of Gastroenterology and Hepatology, New Taipei Municipal TuCheng Hospital, New Taipei City, Taiwan; 4 Department of Neurology, Linkou Chang Gung Memorial Hospital, Taoyuan, Taiwan; 5 Liver Research Center, Linkou Chang Gung Memorial Hospital, Taoyuan, Taiwan; 6 Barts Cancer Institute, Queen Mary University of London, London, UK

**Keywords:** Polypectomy, Delayed post-polypectomy bleeding, Hemoclip, Colon polyp

## Abstract

**Background:**

Delayed post-polypectomy bleeding (PPB) is a major complication of polypectomy. The effect of prophylactic hemoclipping on delayed PPB is uncertain. The aim of this study was to evaluate the effectiveness of prophylactic hemoclipping and identify the risk factors of delayed PPB.

**Methods:**

Patients with polyps sized 6 to 20 mm underwent snare polypectomy from 2015 to 2017 were retrospectively reviewed. The patients with prophylactic hemoclipping for delayed PPB prevention were included in the clipping group, and those without prophylactic hemoclipping were included in the non-clipping group.

The incidence of delayed PPB and time to bleeding were compared between the groups. Multivariate analysis was used to identify the risk factors of delayed PPB. Propensity score matching was used to minimize potential bias.

**Results:**

After propensity score matching, 612 patients with 806 polyps were in the clipping group, and 576 patients with 806 polyps were in the non-clipping group. There were no significant differences in the incidence of delayed PPB and days to bleeding between two groups (0.8% vs 1.3%, *p* = 0.4; 3.4 ± 1.94 days vs 4.13 ± 3.39 days, *p* = 0.94). In the multivariate analysis, the polyp size [Odds ratio (OR):1.16, 95% confidence interval (CI):1.01–1.16, *p* = 0.03), multiple polypectomies (OR: 4.64, 95% CI:1.24–17.44, *p* = 0.02) and a history of anticoagulant use (OR:37.52, 95% CI:6.49–216.8, *p* < 0.001) were associated with delayed PPB.

**Conclusions:**

In polyps sized 6 to 20 mm, prophylactic hemoclip placement did not decrease the risk of delayed PPB. Patients without risk factors including multiple polypectomies and anticoagulant use are no need to performing prophylactic hemoclipping.

## Background

Colorectal cancer (CRC) is a major malignancy worldwide. In 2018, CRC ranked third in terms of incidence and second in terms of mortality with approximately 1,100,000 new cases and 550,000 deaths [1]. Colorectal polyps removal by polypectomy is commonly performed to prevent the development of CRC [2]. However, polypectomy is associated with several complications, such as post-polypectomy bleeding (PPB) and perforation. PPB is the most frequent complication, and can develop during the procedure (immediate PPB) and up to 30 days after the procedure (delayed PPB) [3]. The incidence of delayed PPB has been reported to range from 0.65 to 4.3% in various clinical conditions [4–7]. Although its incidence is low, delayed PPB is associated with severe complications leading to emergency medical interventions, such as hospitalization, blood transfusion, repeat colonoscopic interventions, angiography and even surgical interventions.

Generally, immediate PPB can be well managed by hemoclipping or snare tip soft coagulation with or without the combined use of adrenaline submucosa injection [8, 9]. However, there are no established guidelines for the prevention of delayed PPB. Clinically, prophylactic hemoclipping has been used to reduce the risk of delayed PPB. Nonetheless, the effectiveness of hemoclipping for the prevention of delayed PPB has not been fully established. One retrospective study demonstrated that prophylactic hemoclipping reduced the risk of delayed PPB in colorectal lesions larger than 20 mm [10]. In contrast, another randomized control trial demonstrated that prophylactic hemoclipping does not reduce the incidence of delayed PPB [11]. Moreover, performing prophylactic hemoclipping increases the duration and cost of the procedure. It therefore seems worth examining the effectiveness of prophylactic hemoclipping on delayed PPB, especially in polyps ranging in sizes between 6 and 20 mm, which are mainly resected by snare polypectomy during routine colonoscopy. We designed a case-matched control study with propensity score (PS) matching to compare the delayed PPB outcomes in patients with or without prophylactic hemoclipping.

## Methods

### Patients

This study was approved by institutional review board of Linkou Chang Gung Memorial Hospital (reference No: 201701005B0). Patients undergoing snare polypectomy in the endoscopic therapeutic center of a tertiary medical center in northern Taiwan between Jan 2015 and Jan 2017 were enrolled in the study. The exclusion criteria were as follows: 1) a polyp size ≤5 or > 20 mm, and 2) the presence of immediate bleeding after polypectomy requiring an endoscopic hemostasis procedure. The demography of patients (sex and age), history of antiplatelet (aspirin, clopidogrel) or anticoagulant (warfarin) use, experience of the endoscopists, the occurrence of delayed PPB events, prophylactic hemoclip application and polyp characteristics, including size, morphology and location were recorded. The size of polyps was measured with visual estimation by comparing it with the diameter of the fully open snare loop. The delayed PPB event was defined as the presentation of bloody stools within 14 days after polypectomy, followed by a repeat colonoscopic intervention for hemostasis. The patients who took antiplatelet agent were requested to cease medications for at least 1 week if generally feasible. Heparin bridge therapy was arranged for patients taking anticoagulant medication, and heparin was not administered 6 h before polypectomy. After polypectomy, the use of antiplatelet and anticoagulant medication was resumed on the next day if there was no clinical bleeding symptom and sign.

### Polypectomy procedures

Polypectomies were carried out with a standard colonoscope (Olympus CF Q260AL Tokyo, Japan) and snare (SD-11 U-1; loop diameter, 25 mm; Olympus, Tokyo, Japan). Before snare polypectomies, an adequate volume of normal saline solution was injected into the submucosa beneath the sessile polyps through an injection needle. The polyp was resected by snare with electrosurgical current using an ESG-100 (Olympus, Tokyo, Japan) with cutting and coagulation settings ranging between 20 W and 40 W. After the polypectomy procedure, hemoclips (EZClip, Olympus, Tokyo, Japan) were prophylactically placed according to decision of the polypectomy-performed endoscopist. The endoscopic images of prophylactic hemoclip placement were shown in supplementary figure. In patients with major bleeding after polypectomy, various hemostatic procedures including hemoclipping were performed and these patients were excluded from this study. Carbon dioxide was used throughout the polypectomy procedures in all patients.

### Propensity score matching

**Fig. 1:**
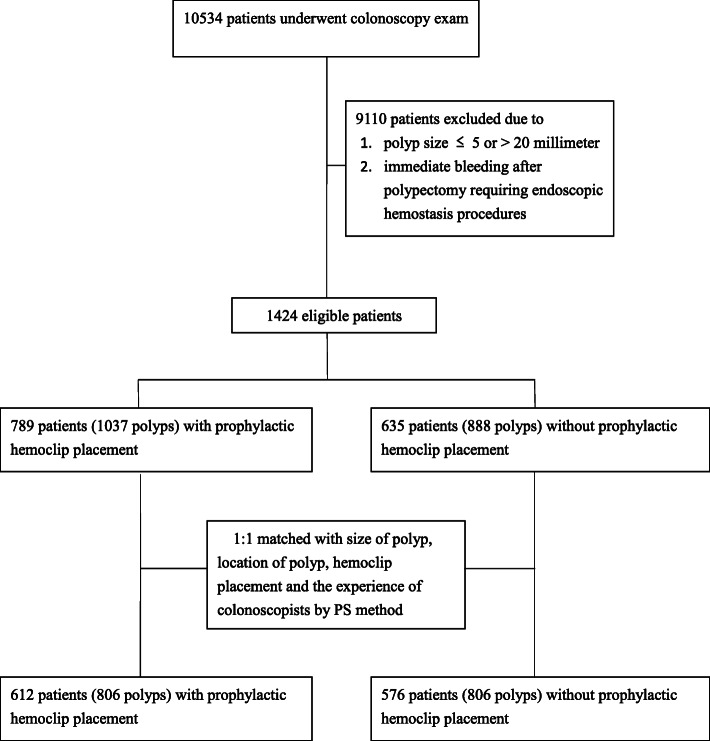
Flow chart of patient selection

To minimize the risk of bias and balance the significant differences between the patients with or without prophylactic hemoclipping, a PS matching analysis was performed. The propensity score of each participant was calculated using the following categorical variables: size of the polyp, location of the polyp, hemoclip placement and experience of the endoscopists. After PS generation, the patients with prophylactic hemoclipping and those without prophylactic hemoclipping underwent 1:1 nearest available PS matching. Both clipping and non-clipping patients who did not meet the matching criteria were excluded. The patient selection flow chart is shown in Fig. 1.

### Statistical analyses

Statistical analyses were performed using SPSS 22 (IBM Corp., Armonk, NY, USA) for Windows. The descriptive statistics and frequency distributions were calculated. All continuous data are shown as mean values and SD, and the categorical variables are presented as absolute and relative frequencies. The data were analyzed by using either the Mann-Whitney U test for the continuous variables or the chi-square test for the categorical variables. Univariate and multivariate logistic regressions were used to examine the associations between prophylactic hemoclip placement and delayed PPB events. Odds ratios (ORs) and 95% confidence intervals (CIs) were calculated. Statistical significance was defined as a *p* value < 0.05.

## Results

### Baseline characteristics of the patients before and after PS matching

A total of 1925 polyps in 1424 patients were included in this study. The studied patients were divided into the clipping and non-clipping groups based on the application of prophylactic hemoclipping after polypectomy. The average number of polypectomies per patient was 1.35. The clipping group included 789 patients with 1037 polyps, and the non-clipping group included 635 patients with 888 polyps. The original demographics and characteristics of the patients are shown in Table 1. The polyp size in the clipping group was significantly larger than that in the non-clipping group (10.09 ± 3.45 mm vs 9.10 ± 3.14 mm; *p* < 0.001). In the non-clipping group, the prevalence of polyps in the distal colon was significantly higher than that in the clipping group (61.6% vs 52.5%; *p* < 0.001). In the patients with multiple polypectomies, the proportion of hemoclip usage was significantly lower than that in the patients with a single polypectomy (41.8% vs 49.7%; *p* < 0.001). After PS matching, the clipping group included 612 patients with 806 polyps, and the non-clipping group included 576 patients with 806 polyps. The baseline characteristics were comparable between the matched groups, and there were no significant differences in the polyp size, polyp location and endoscopist’s experience (Table 1).

**Table 1:** Demographic and clinical characteristics of the patients before and after propensity score matching, n (%)

	Before PSM (*N* = 1424)			After PSM (*N* = 1188)		
	Clipping group	Non-clipping group	*p* value	Clipping group	Non-clipping group	*p* value
	*N* = 789 (55.0%) 1037 polyps (53.8%)	*N* = 635 (45.0%) 888 polyps (46.2%)		*N* = 612 (51.5%); 806 polyps	*N* = 576 (48.5%); 806 polyps	
**Gender**/Male	572 (72.5)	439 (69.1)	0.164	445 (72.7)	402 (69.8)	0.266
**Age** (Mean ± S.D.)	59.73 ± 11.66	60.43 ± 11.57	0.192	59.63 ± 11.75	60.46 ± 11.64	0.200
**Polyp size (mm)** (Mean ± S.D.)	10.09 ± 3.45	9.10 ± 3.14	< 0.001	9.32 ± 3.16	9.36 ± 3.18	0.845
**Polyp location**			< 0.001			0.800
Proximal	493 (47.5)	345 (38.9)		332 (41.2)	327 (40.6)	
Distal	544 (52.5)	543 (61.1)		474 (58.8)	479 (59.4)	
**Polyp shape**			0.927			0.136
Pedunculated	134 (12.9)	134 (12.9)		93 (11.5)	113 (14.0)	
Sessile	903 (87.1)	903 (87.1)		713 (88.5)	693 (86.0)	
**Endoscopist experience**			< 0.001			0.454
< =300	384 (37.0)	249 (30.9)		263 (32.6)	249 (30.9)	
> 300	653 (63.0)	557 (69.1)		543 (67.4)	557 (69.1)	
Antispastic agent	938 (90.5)	786 (88.5)	0.165	729 (90.4)	714 (88.6)	0.223
Multiple polypectomies	433 (41.8)	441 (49.7)	0.001	323 (40.1)	401 (49.8)	< 0.001
Conscious sedation	745 (71.8)	627 (70.6)	0.551	575 (71.3)	564 (70.0)	0.547
Antiplatelet use history	46 (4.4)	28 (3.2)	0.197	35 (4.4)	26 (3.2)	0.360
Anticoagulant use history	6 (0.6)	9 (1.0)		5 (0.6)	8 (1.0)	
No medication use	985 (95.0)	851 (95.8)		766 (95.0)	722 (95.8)	

*PSM* propensity score matching; *S.D.* standard deviation

### Delayed post-polypectomy bleeding

**Fig. 2:**
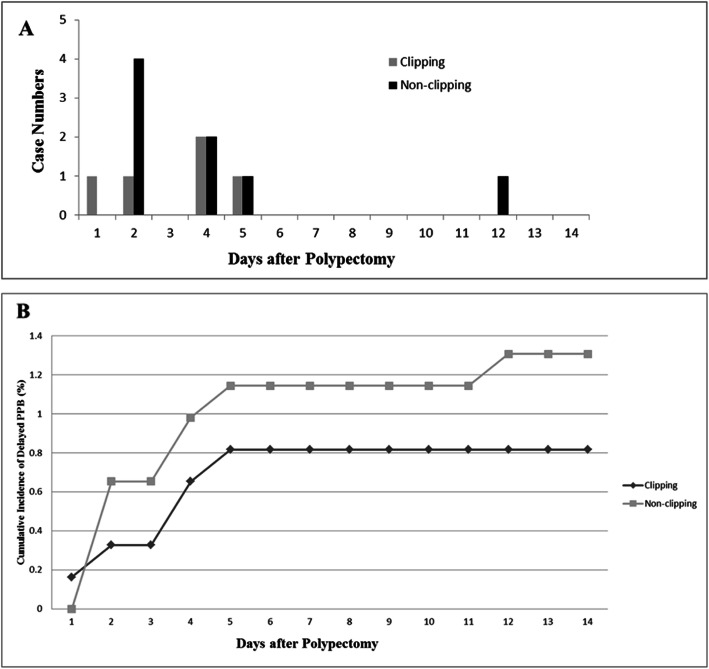
Case numbers (**a**) and cumulative incidences (**b**) of delayed PPB in clipping and no-clipping groups

Before PS matching, the delayed PPB events occurred in 9 (1.1%) patients in the clipping group and 8 (1.3%) patients in the non-clipping group, and there was no significant difference between the groups (*p* = 0.93) (Table 2). The mean days to bleeding were 3.44 ± 1.74 days in the clipping group and 4.13 ± 3.40 days in the non-clipping group. There was no significant difference in time to bleeding between the groups (*p* = 0.963). After PS matching, PPB events occurred in 5 (0.8%) patients in the clipping group and 8 (1.3%) patients in the non-clipping group, and there was no significant difference between the groups (*p* = 0.4). The mean days to bleeding were 3.4 ± 1.94 days in the clipping group and 4.13 ± 3.39 days in the non-clipping group. There was no significant difference in the time to bleeding between the groups (*p* = 0.943). Figure 2 shows the case numbers and cumulative incidence of delayed PPB in both groups after PS matching.

**Table 2:** Clinical outcomes in the clipping and non-clipping groups before and after propensity score matching

	Before PSM (N = 1424)			After PSM (N = 1188)		
	Clipping group	Non-clipping group	*p* value	Clipping group	Non-clipping group	*p* value
	N = 789 (55.0%) 1037 polyps (53.8%)	N = 635 (45.0%) 888 polyps (46.2%)		N = 612 (51.5%); 806 polyps	N = 576 (48.5%); 806 polyps	
Bleeding event, No (%)	9 (1.14)	8 (1.25)	0.938	5 (0.81)	8 (1.38)	0.403
Days to bleeding (mean ± S.D.)	3.44 ± 1.74	4.13 ± 3.40	0.963	3.4 ± 1.94	4.13 ± 3.39	0.943

*PSM* propensity score matching; *S.D.* standard deviation

### Risk factors of delayed post-polypectomy bleeding

To evaluate the risk factors of delay PPB after PS matching, univariate and multivariate analyses were performed. The results of the analyses are shown in Table 3. In the univariate analysis, polyp size was a risk factor for delayed PPB (OR: 1.14, 95% CI: 1.00–1.3, *p* = 0.04), indicating a 14% increased risk per millimeter increase in size. Multiple polypectomies and a history of anticoagulant use were also associated with delayed PPB (OR: 4.13, 95% CI: 1.13–15.06, *p* = 0.03; OR: 25.24, 95% CI: 4.99–127.4, *p* < 0.001, respectively). In the multivariate analysis, considering factors, such as sex, the polyp size, polypectomy times and anticoagulant use history, the polyp size, multiple polypectomies and anticoagulant use history were associated with delayed PPB (OR:1.16, 95% CI:1.01–1.16, *p* = 0.03; OR: 4.64, 95% CI:1.24–17.44; *p* = 0.02; OR:37.52, 95% CI:6.49–216.8, *p* < 0.001, respectively).

**Table 3:** Logistic regression analysis for delayed PPB predictors after propensity score matching

	Univariate OR	95% CI	*p* value	Multivariate OR	95% CI	*p* value
Age (> = 65:< 65)	1.24	0.40–3.80	0.70	N/A	N/A	N/A
Male (female)	0.56	0.24–1.30	0.18	0.37	0.11–1.22	0.10
Polyp size (increasing 1 mm)	1.14	1.00–1.30	**0.04**	1.16	1.01–1.16	**0.03**
Polyp location (vs distal colon)	1.69	0.56–5.06	0.34	N/A	N/A	N/A
Polyp shape (vs sessile)	2.06	0.56–7.55	0.27	N/A	N/A	N/A
Prophylactic hemoclips	0.40	0.20–1.91	0.93	N/A	N/A	N/A
Endoscopist experience (< 300 polypectomies)	2.57	0.56–11.66	0.22	N/A	N/A	N/A
Multiple polypectomies	4.13	1.13–15.06	**0.03**	4.64	1.24–17.44	**0.02**
Antiplatelet use history	–	–	0.99	N/A	N/A	N/A
Anticoagulant use history	25.24	4.99–127.4	**< 0.001**	37.52	6.49–216.8	**< 0.001**

*OR* odds ratio; *CI* confidence interval

### Subgroup analysis of delayed post-polypectomy bleeding

**Fig. 3:**
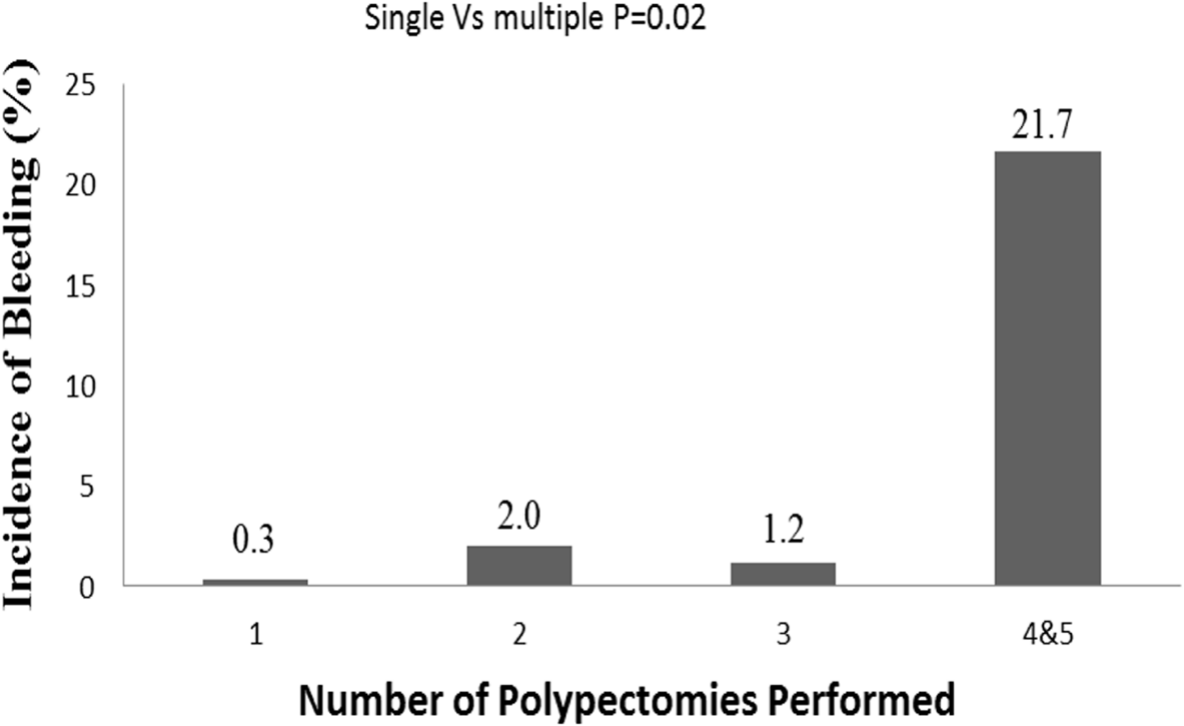
Incidences of delayed PPB in relation to the numbers of polypectomies performed in a one-time colonoscopy session

Because the patients with multiple polypectomies were considered at a greater risk of PPB, further subgroup analysis was performed. The incidences of bleeding in relation to the numbers of polypectomies performed in a one-time colonoscopy session are shown in Fig. 3. In the patients with a single polypectomy, the incidence of bleeding was 0.3% (3/888). The incidence of bleeding was 2.0% (4/198) in the patients with two polypectomies, 1.2% (1/79) in the patients with three polypectomies and 21.7% (5/23) in the patients with four and five polypectomies. The average incidence of bleeding in the patients with ≥2 polypectomies was 3.3%, which was significantly higher than that in the patients with a single polypectomy (*p* = 0.02) (Fig. 3). A multivariate analysis was performed to evaluate the risk of delayed PPB in the patients with single or multiple polypectomies in a single colonoscopy procedure, and the results of the multivariate analysis are shown in Table 4. In the patients with a single polypectomy, the polyp size was associated with delayed PPB (OR: 1.30; 95% CI: 1.02–1.65; *p* = 0.03). In the patients with multiple polypectomies during a single colonoscopy procedure, the female sex and a history of anticoagulant use were associated with delayed PPB. (OR: 6.35, 95% CI: 1.49–27.06, *p* = 0.01; OR: 86.1, 95% CI: 11.13–665.9, *p* < 0.001).

**Table 4:** Multivariate analyses of delayed PPB in the single and multiple polypectomies subgroups

	Single polypectomy group			≥ 2 polypectomies group		
	Multivariate OR	95% CI	*p* value	Multivariate OR	95% CI	*p* value
Female (male)	–	–	–	6.35	1.49–27.06	0.01
Polyp size (increasing 1 mm)	1.30	1.02–1.65	0.03	1.10	0.92–1.32	0.29
Anticoagulant use history	–	–	–	86.1	11.13–665.9	< 0.001

*OR* odds ratio; *CI* confidence interval

## Discussion

In this study, we demonstrated that prophylactic hemoclip placement could not decrease the incidence of delayed PPB in polyps between 6 and 20 mm. In addition, we demonstrated that the polyp size, multiple polypectomies, and a history of anticoagulant use in a single colonoscopy procedure were associated with delayed PPB. The incidence of delayed bleeding in the patients with multiple polypectomies was significantly higher than that in those with a single polypectomy. In the patients with a single polypectomy, the polyp size was associated with delayed PPB, while the female sex and a history of anticoagulant use were associated with delayed PPB in the patients with multiple polypectomies in a single colonoscopy procedure.

Hemoclip placement is a useful modality for treating immediate PPB. However, the effects of prophylactic hemoclip placement on delayed PPB are uncertain. One multi-center randomized control trial found that prophylactic hemoclip placement after resection of non-pedunculated polyps ≥2 cm in the proximal colon reduces the risk of post-polypectomy bleeding [12]. In contrast, one randomized control trial conducted in Japan and one case-control study conducted in the United States revealed that prophylactic hemoclipping could not decrease the incidence of delayed PPB in the polyps with small and intermediate size [11, 13]. In both studies, however, the case numbers were less than 300, and the studies were relatively small. In our study, we demonstrated a similar incidence of delayed PPB in the clipping and non-clipping groups with greater case numbers with polyps sized between 6 and 20 mm. Recently, one randomized control trial conducted in the United States showed a similar result to our study [14]. However, the patient population was predominately male (> 95%), and whether their finding applies to female patients is uncertain. Although colorectal polyps is a male predominate disease [15], the female proportion in our study is approximately 30%, which is much higher than that in the previous study. Therefore, our study has a more balanced patient distribution leading to practical results based on clinical experience.

Many factors associated with an increase in delayed PPB are related to the polyp characteristics, such as the size and location, or the patients’ underlying health status, such as an age < 50 years and the presence of hypertension [5, 16–18]. In two nationwide surveys investigating colonoscopic polypectomy complications in Germany and the United Kingdom, the polyp size played an important role in the prediction of delayed PPB [4, 19]. Clearly, larger polyps resulted in larger post-polypectomy wounds, leading to a higher bleeding incidence. Our results concurred with this finding. In general, large polyps sized ≥2 cm are recommended for removal by endoscopic mucosal resection (EMR) to achieve complete resection, and the incidence of delayed PPB could be significantly lowered by prophylactic hemoclip placement [10]. In contrast, when the polyp size is small or intermediate, our results revealed that the incidence of delayed PPB and the days to bleeding do not decrease following prophylactic hemoclip placement. Thus, after the removal of polyps sized 6 to 20 mm, there is no need to perform prophylactic hemoclipping, thus avoiding the unnecessary medical cost of hemoclips and the procedure time involved in hemoclipping.

Multiple polyps may be found in one individual during colonoscopy, necessitating multiple polypectomies in a single colonoscopy procedure. Our study demonstrated that the incidence of delayed PPB in patients with multiple polypectomies during a single colonoscopy procedure was significantly higher than that in patients with a single polypectomy, and multiple polypectomies were significantly associated with delayed PPB. Moreover, we demonstrated that the polyp size was associated with delayed PPB in a single polypectomy, while the female sex and a history of anticoagulant use were associated with delayed PPB in patients with multiple polypectomies. Interestingly, in patients with multiple polypectomies, the polyp size was not a risk factor of delayed PPB. Although the cause of increased incidence of delayed PPB in multiple polypectomies is unclear, we presume that more time might be needed for the wound healing process in patients with multiple polypectomies. In addition, our result demonstrated that patient-related factors, such as female sex and a history of anticoagulant may have a greater effect on post-polypectomy wound healing process in patients with multiple polypectomies. The ORs of delayed bleeding in female patients with multiple polypectomies was 6.35 compared to that in male patients. The reason why delayed bleeding complicated in female sex and only in multiple polypectomies rather than single polypectomy was unclear. Further studies regarding the exact mechanism of this finding are needed.

Although all patients taking anticoagulant agents were given heparin bridge therapy and heparin was hold 6 h prior to the polypectomy procedure, a history of anticoagulant use was still associated with delayed PPB. Our finding is consistent with a previous study [20]. Additionally, these patients were arranged heparin bridge therapy and several studies demonstrated that heparin bridge therapy is associated with delayed PPB [21–23]. Interestingly, the effect of anticoagulant or heparin therapy is associated with delayed PPB in patients with multiple polypectomies rather than single polypectomy. The ORs of delayed PPB in patients with a history of anticoagulant agent use underwent multiple polypectomies was 86.1 compared to that in patients without anticoagulant agent use. Clinically, endoscopists should identify this high risk group, especially when performing multiple polypectomies in a single colonoscopy.

Our study had several limitations. First, this study was a retrospective study; therefore, some confounding factors and selection bias might have influenced our results. However, PS matching was used to minimize the effect of selection bias and render the variables comparable between the clipping and non-clipping groups. Second, the underlying comorbidity of the patients was not analyzed in our study. However, previous studies have revealed that delayed PPB is not associated with common major systemic and organ-specific diseases, such as diabetes mellitus, chronic kidney disease, chronic liver disease and cardiovascular disease [17, 18]. Third, although the patients were instructed to discontinue the use of antiplatelet medication 7 days prior to the polypectomy date, the exact duration of discontinuation was unclear. Fourth, the polyp size was measured with visual estimation by comparing it with the diameter of the fully open snare loop. The measurement may be inaccurate and the interobserver variability regarding the polyp size may be wide. Finally, the immediate bleeding is an independent risk factor in a prospective study but is not analyzed in our study [17]. Generally, if immediate bleeding occurred, variable methods such as hemoclipping, snare-tip coagulation, adrenaline submucosa injection or endoloop placement would be used for hemostasis. Therefore, the effect of hemoclipping would be difficult to evaluate.

## Conclusion

Prophylactic hemoclip placement did not decrease the incidence of delayed PPB after the removal of polyps sized 6 to 20 mm. Patients without risk factors including multiple polypectomies and anticoagulant use are no need to performing prophylactic hemoclipping.

## Supplementary information

**Additional file 1: Supplementary figure.** Steps of polypectomy and prophylactic hemoclip placement. (A) Polyp elevated with submucosal injection (B) Wound after polypectomy (C) Wound closure by prophylactic hemoclip.

## Data Availability

The data used to support the findings of this study are available from the corresponding author upon request.

## Abbreviations

PPB: Post-polypectomy bleeding
OR: Odds ratio
CI: Confidence interval
CRC: Colorectal cancer
PS: Propensity score
EMR: Endoscopic mucosal resection
