# Supplementary material for: The effect of prophylactic hemoclip placement and risk factors of delayed post-polypectomy bleeding in polyps sized 6 to 20 millimeters: a propensity score matching analysis
Source: BMC Gastroenterol. 2020 Sep 22;20:309. doi: 10.1186/s12876-020-01454-1 (PMC7510104; doi:10.1186/s12876-020-01454-1)
Supplement: Supplementary file 1 — Additional file 1: Supplementary figure. Steps of polypectomy and prophylactic hemoclip placement. (A) Polyp elevated with submucosal injection (B) Wound after polypectomy (C) Wound closure by prophylactic hemoclip. [file 12876_2020_1454_MOESM1_ESM.doc]

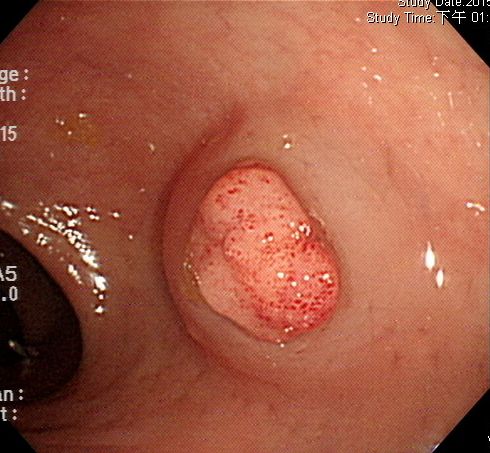

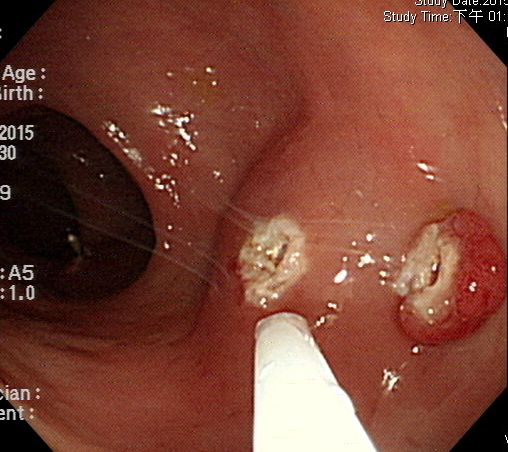

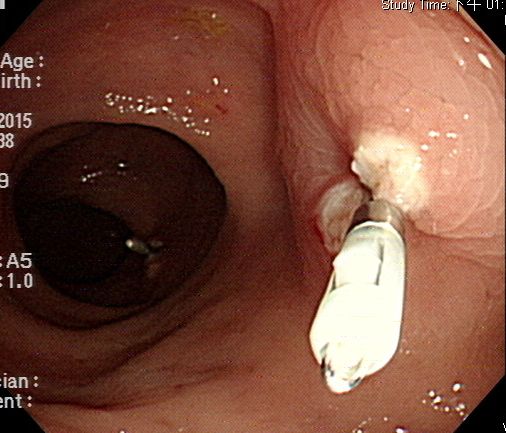


C

B

A

Supplementary figure. Steps of polypectomy and prophylactic hemoclip placement.

(A) Polyp elevated with submucosal injection (B) Wound after polypectomy (C) Wound closure by prophylactic hemoclip
